# Cardiac MRI with late gadolinium enhancement shows cardiac involvement 3–6 months after severe acute COVID-19 similar to or worse than PIMS

**DOI:** 10.3389/fcvm.2023.1115389

**Published:** 2023-01-25

**Authors:** Lyubov A. Chochkova-Bukova, Dominik Funken, Mila Bukova, Kamelia Z. Genova, Sadika Ali, Snezhana Stoencheva, Ivanka N. Paskaleva, Zeira Halil, Ivelina Neicheva, Anastasia Shishmanova, Kristina Stefanova Kelly, Ivan S. Ivanov

**Affiliations:** ^1^Department of Pediatrics and Medical Genetics, Medical University Plovdiv, Plovdiv, Bulgaria; ^2^Department of Pediatric Pneumology, Allergy and Neonatology, Hannover Medical School, Hannover, Germany; ^3^Department of Pediatric Cardiology and Pediatric Intensive Care, Hannover Medical School, Hannover, Germany; ^4^Clinic of Imaging Diagnostics, University Hospital “N. I. Pirogov”, Sofia, Bulgaria

**Keywords:** cardiac imaging, echocardiography, SARS-CoV2, heart involvement, Multisystem Inflammatory Syndrome in Children (MIS-C)

## Abstract

**Background:**

Coronavirus disease 2019 (COVID-19) in children is rarely severe. However, severe courses occur, especially in the presence of risk factors. A minority of children develop pediatric inflammatory multisystem syndrome (PIMS) with substantial morbidity. While the importance of cardiac involvement after PIMS is well established, its role after severe acute COVID-19 remains unclear. We aim to compare cardiac sequelae of children after severe acute COVID-19 using cardiac MRI and compare them with patients after PIMS.

**Methods:**

For this prospective cohort study, we recruited patients with acute COVID or PIMS in a single center. Clinical follow-up, lab work, ECG, and echocardiography were done within 2 days after disease onset and 3–6 months after discharge. At the last visit 3–6 months later, cardiac MRI (CMR) with late gadolinium enhancement (LGE) was performed to evaluate cardiac sequelae and compare both groups.

**Results:**

Data were obtained from *n* = 14 patients with PIMS and *n* = 7 patients with severe acute COVID-19. At the start of the respective disease, left ventricular (LV) ejection fraction was reduced in seven patients with PIMS but none in the acute COVID-19 group. Transient mitral valve insufficiency was present in 38% of patients, of whom PIMS accounted for 7/8 cases. Eight patients (38%) with PIMS presented coronary artery abnormalities, with normalization in 7/8 patients. A significant decrease in LV mass index 3–6 months after disease onset was observed in both groups. MRI follow-up revealed non-ischemic myocardial pattern of LGE in 12/21 patients- in all (6/6) after severe acute COVID-19 and in less than half (6/14) after PIMS. Normal body weight-adjusted stroke volumes and end-diastolic volumes were found in 20/21 patients.

**Conclusions:**

We show that children suffering from severe acute COVID-19 have a similar, or worse, cardiac risk profile as patients with PIMS. Both patient groups should therefore receive close pediatric cardiac follow-up examinations. Cardiac MRI is the technique of choice, as most patients presented with delayed LGE as a sign of persistent cardiac injury despite normalization of laboratory and echocardiographic findings.

## 1. Introduction

Coronavirus disease 2019 (COVID-19) usually has mild or no symptoms in children and adolescents. However, severe courses may occur, especially in patients with pre-existing risk factors such as chronic underlying disease or obesity (1). Pediatric Inflammatory Multisystem Syndrome (PIMS), also referred to as Multisystem Inflammatory Syndrome in children (MIS-C), is a severe acute inflammatory disease affecting various organs. PIMS usually manifests 2–8 weeks after coronavirus infection in children. The condition is rare but severe and its pathophysiology is still not well understood. However, numerous reports have led to a complete characterization of its clinical features, but a misdirected and overshooting immune response is suspected as a predisposition. Data on secondary damage to the cardiovascular system varies depending on the utilized techniques, parameters, and follow-up period. Results range from complete remission to 36% measurable long-term damage (2–5).

Despite significant clinical overlap between severe acute COVID-19 and PIMS, some peculiarities in presentation patterns and organ involvement may help their differentiation. Severe respiratory symptoms are more frequently seen in severe acute COVID-19, whereas severe cardiac involvement is more common in PIMS (6). Data on secondary damage to the cardiovascular system varies depending on the utilized techniques, parameters, and follow-up period. Results range from complete remission to 36% measurable long-term damage (2, 10).

Although SARS-CoV2 infection usually has a benign course, the presence of risk factors such as pre-existing chronic disease and adiposity may imply a severe disease in pediatric patients. In the United States, up to one-third of children with COVID-19-related hospitalization were reported to have severe disease (7–9). While the importance of cardiac involvement after PIMS is well established, its magnitude after severe acute COVID-19 remains unclear. A cardiac MRI screening study in adult patients who recovered from COVID-19 suggested that up to 60% of COVID-19 survivors had developed myocarditis, regardless of disease severity (10). This is especially concerning from a pediatric point of view, as most pediatric SARS-CoV2 cases are mild or asymptomatic. However, there is increasing evidence that myocarditis may also develop after pediatric COVID-19 infection. These children are at risk of significant but unnoticed cardiac sequelae (10–12). Cardiac MRI is the reference standard for the evaluation of myocardial structure and function in the follow-up of myocarditis (13). During the acute or subacute phase, areas of LGE have been established as a sign of necrosis and fibrosis of the myocardium. Myocardial edema is usually identified and qualitatively assessed on TIRM images. The presence of a prolonged T2 relaxation time is an additional sign of increased myocardial water content. The present prospective study aims to compare children's cardiac sequelae after severe acute COVID-19 and after PIMS.

## 2. Materials and methods

This is a monocentric study conducted prospectively from September 2021 until February 2022 at the Department of Pediatrics and Medical Genetics, Medical University Plovdiv. Legal consent was obtained from the patient's guardians and patients older than 16 years. Analyses were conducted in accordance with the local institutional review board of Plovdiv Medical University Scientific Ethics Committee (P-2501/2022_RKNE_F6EAA81C7D).

The inclusion criteria for the study were severe acute COVID-19 infection or PIMS according to the Center for Disease Control (CDC, US) criteria for pediatric patients published in May 2020 (14). Patients with pre-existing heart conditions, sepsis, or biological treatment were excluded from this study.

Polymerase chain reaction (PCR) from nose and throat swabs and/or serology for SARS-CoV-2 was performed on all cases suspected of acute COVID-19 or PIMS. Positive cases were screened to fulfill the criteria for severe acute SARS-CoV2 infection. Children presenting with fever, clinical and biochemical inflammation features, and single or multiorgan dysfunction were screened for possible PIMS.

The PIMS group was further evaluated for fulfilling complete or incomplete Kawasaki disease (KD) criteria and for hemodynamic stability. Based on those criteria, patients were divided into 4 groups- KD/KD-like or no-KD with or without hemodynamic shock. Disease severity was assessed for all patients using the PEdiatric Logistic Organ Dysfunction score 2 (PELOD2) (15). Chest radiographs, CT scans, abdominal ultrasounds, and other investigations were performed as clinically indicated. Patients were monitored continuously and received daily ECG and echocardiography checks. After clinical stabilization, the frequency of examinations was reduced to as clinically required. Clinical laboratory tests were performed regularly. Results after admission and 3–6 months later were analyzed for this study.

### 2.1. Cardiac monitoring

#### 2.1.1. ECG and echocardiography

ECGs were performed routinely. Echocardiograms included 2-dimensional, Doppler, and M-mode modalities. For this study, we compared an initial examination within the first 2 days of hospitalization with a follow-up examination 3–6 months after discharge. Global left ventricular (LV) systolic function was assessed with a 2D method (16). Left ventricular ejection fraction (LVEF) was categorized based on the Teichholtz and Simpson EF methods. Normal values for EF in children were between 56 and 78%. EF was classified as normal (EF ≥ 55%), slightly reduced (EF 41–55%), moderately reduced (EF 31–40%), and markedly reduced (EF ≤ 30%) (11). Mitral regurgitation was assessed qualitatively and semiquantitatively according to the mitral jet and its components in color Doppler mode. Mitral regurgitation was graded as mild, moderate, or severe based on the size and extent of the color-flow Doppler signal into the left atrium, left atrial and left ventricular size, respectively. Coronary artery measurements were from inner to inner edge. *Z*-scores were calculated for left ventricular end-diastolic diameter (LVEDD), interventricular septal thickness (IVS), left ventricular posterior wall thickness in diastole (LVPW) (16), coronary artery (CA) diameter (17), and left ventricular mass (18). Left ventricular mass was estimated using the Devereux equation. Left ventricular mass index (LVMI) was calculated with body surface area.

#### 2.1.2. Cardiac MRI

MRI was performed between 3 and 6 months after disease onset using a 1,5 Tesla whole-body scanner (Siemens Magnetom Aera, Erlangen, Germany) according to a standardized protocol. This included modules for the evaluation of cardiac morphology, function, contractility, and tissue characteristics of changes in the myocardium using T1 and T2 mapping for quantification.

Areas of myocardial edema were assessed on T2/turbo inversion recovery magnitude (TIRM) images. A signal intensity ratio of myocardium/skeletal muscle of ≥2.0 was classified as abnormal, indicating myocardial edema. The type of late gadolinium enhancement (LGE) was classified according to its location in endocardial, intramural, epicardial, or transmural type. LGE images were evaluated by phase-sensitive inversion recovery (PSIR). Values of T1 and T2 relaxation times were measured in areas with edema and/or LGE and in the unaffected myocardium. The threshold for abnormal relaxation time was >1,100 ms for T1 and >49 ms for T2 images, respectively. Normal values were determined on 20 healthy age-matched volunteers with no history of cardiovascular disease who received chest MRIs in the same center before the beginning of this study in September 2021.

The volumes and function of both ventricles were calculated using the ARGUS software (Siemens, Erlangen, Germany). Semi-automatic delineation of endocardial and epicardial contours of the myocardium of both ventricles was manually corrected for each image in end-systole and end-diastole. All volumes and masses were normalized to the body surface area of the respective patient.

### 2.2. Statistical analysis

Statistical analysis and visual representation of data were performed using the statistical programming language R Version 1.3.1093 (https://www.r-project.org/). Non-parametrical Mann–Whitney test was used for comparison of non-dependent groups, Wilcoxon signed-rank test was used for dependent comparisons. Statistical significance was defined at a *p*-value of < 0.05.

## 3. Results

### 3.1. Cohort characterization

During the 6-month study period, 21 patients were recruited. The median age was of 12.0 years (5.0–18.0 years). Fourteen out of 21 patients (67%) were male. All patients were of Caucasian descent.

One-third (7/21) of the patients suffered from severe acute COVID-19, and 14 met the criteria for PIMS. Among the 14 patients classified as PIMS, 11 showed complete or incomplete criteria of Kawasaki's disease, and the other three suffered from no-KD PIMS with cardiovascular shock as major presentation. No patients with severe acute COVID-19 developed PIMS during *f* /*u*, and no patients with PIMS had a history of prior symptomatic severe SARS-CoV2 infection.

All 21 patients presented with pyrexia with a median fever duration of 8 days. The 5 other most common symptoms were respiratory (81%), gastrointestinal (62%), conjunctivitis (52%), sore throat (52%), and hepatosplenomegaly (48%) (Table 1). Five patients with PIMS had acute kidney injury.

**Table 1:** Cohort characterization.

		**Severe acute COVID-19**	**PIMS**
**Number of cases**		***n*** = **7**	***n*** = **14**
Sex	Male	6 (86%)	8 (57%)
	Female	1 (14%)	6 (43%)
Age [median]	Years	12	12
Risk factors	Adipositas (BMI > 30)	4 (57%)	1 (7%)
	Pre-existing medical condition	1 (14%)	2 (14%)
Echocardiography	Normal	7 (100%)^*^	14 (100%)
	Pathologic (EF, coro, effu, MI)	0	0
Cardiac MRI	Nomal	0	7 (50%)
	Pathologic	6 (100)^**^	7 (50%)
ECG	Normal	6 (86%)	13 (93%)
	Pathologic	1 (14%)	1 (7%)
Clinical symptoms	Persistent fever [median duration]	100% [8 days]	100% [8 days]
	GI symptoms	1 (14%)	12 (86%)
	Rash	0	9 (64%)
	Conjunctivitis	0	11 (79%)
	Neurolog. symptoms	2 (28%)	4 (29%)
	Respiratory symptoms	7 (100%)	10 (71%)
	Sore throat	3 (43%)	8 (57%)
	Myalgia	0	4 (29)
	Swollen hands/feet	0	8 (57%)
	Lymphadenopathy	0	8 (57%)
	Kawasaki criteria complete	na	5 (36%)
	HSM	0	10 (71%)

Characteristics of 21 pediatric patients with severe acute COVID-19 or pediatric inflammatory multisystem syndrome (PIMS). BMI, body mass index. Pre-existing medical conditions were Sturge-Weber-syndrome in one patient with acute COVID-19 and two patients in the PIMS group suffering from thalassemia and asthma or B cell leukemia, respectively. Pathologic echocardiography was defined as at least one of the four criteria (1) pericardial effusion, (2) reduced ejection fraction, (3) coronary anomaly, or (4) mitral valve regurgitation.

* In four patients, due to adiposity, the body surface area was >2 m^2^ resulting in an inapplicability of pediatric z-scores. All values were normal according to adult criteria and therefore considered normal. MRI, magnetic resonance imaging.

** No MRI data available in one patient with acute COVID-19. ECG, electrocardiogram; GI, gastrointestinal; Neurolog., neurological, consisting of headache, altered consciousness, meningoencephalitis, and encephalopathy; HSM, hepatosplenomegaly.

All except one of our patients showed hypoxemia and received oxygen therapy. Two patients suffering from acute COVID-19 needed respiratory support- one on invasive ventilation and one on high-flow. Most children (18/21) received 10 L/min oxygen *via* facemask with oxygen reservoir bag. The PaO_2_/FiO_2_ (P/F) ratio ranged from 60 to 448, with only four patients with PIMS over the ARDS threshold of 300.

Ten patients with PIMS (8 with KD-like presentation) had cardiovascular shock and received inotropes and vasopressors. No patients with severe acute COVID-19 needed catecholamines. No patients needed extracorporeal life support, and all patients were discharged alive and in an improved condition.

All patients with severe acute COVID-19 received a low PELOD 2 score of 0–1 points except one patient with 4 points. Scores in the PIMS groups were also mild, ranging from 0 to 5 points. There was no correlation between severity score and cardiac involvement (Supplementary material).

### 3.2. Clinical laboratory results

All patients showed elevated inflammatory markers (leukocytosis, elevated C-reactive protein, and ferritin). As expected, elevation of cardiac disease markers (troponin T, pro-BNP) was more profound in the PIMS group but normalized in all patients after 3 months (**Figure 2**). Laboratory results did not correlate with MRI findings or disease severity (Supplementary Figures 1, 2).

### 3.3. Imaging

#### 3.3.1. Electrocardiography and echocardiography

ECG abnormalities were present in 5 (24%) and normalized in all patients after 3–6 months.

Left ventricular ejection fraction (LVEF) was reduced in seven patients with PIMS but none in the acute COVID-19 group. The reduction of LVEF resolved in all patients. Interestingly, only one patient with acute COVID-19 showed reduced LVEF during follow-up examination at 3–6 months.

The left ventricular mass index did not differ in patients with COVID-19 or PIMS at the time of disease onset. Both groups showed a significant reduction in LVMI in the follow-up examination (Supplementary Figure 3). Transient mitral valve insufficiency (MI) was present in 8 (38%) of patients, 7 of them with PIMS. While most patients with PIMS recovered, we found newly developed mild MI during follow-up in 2 patients, one in each group.

Eight patients (38%) with PIMS presented coronary artery dilatation, with normalization in 7/8 patients. One patient with PIMS showed persistent coronary dilatation after 6 months of follow-up.

#### 3.3.2. Cardiac MRI

**Figure 1:**
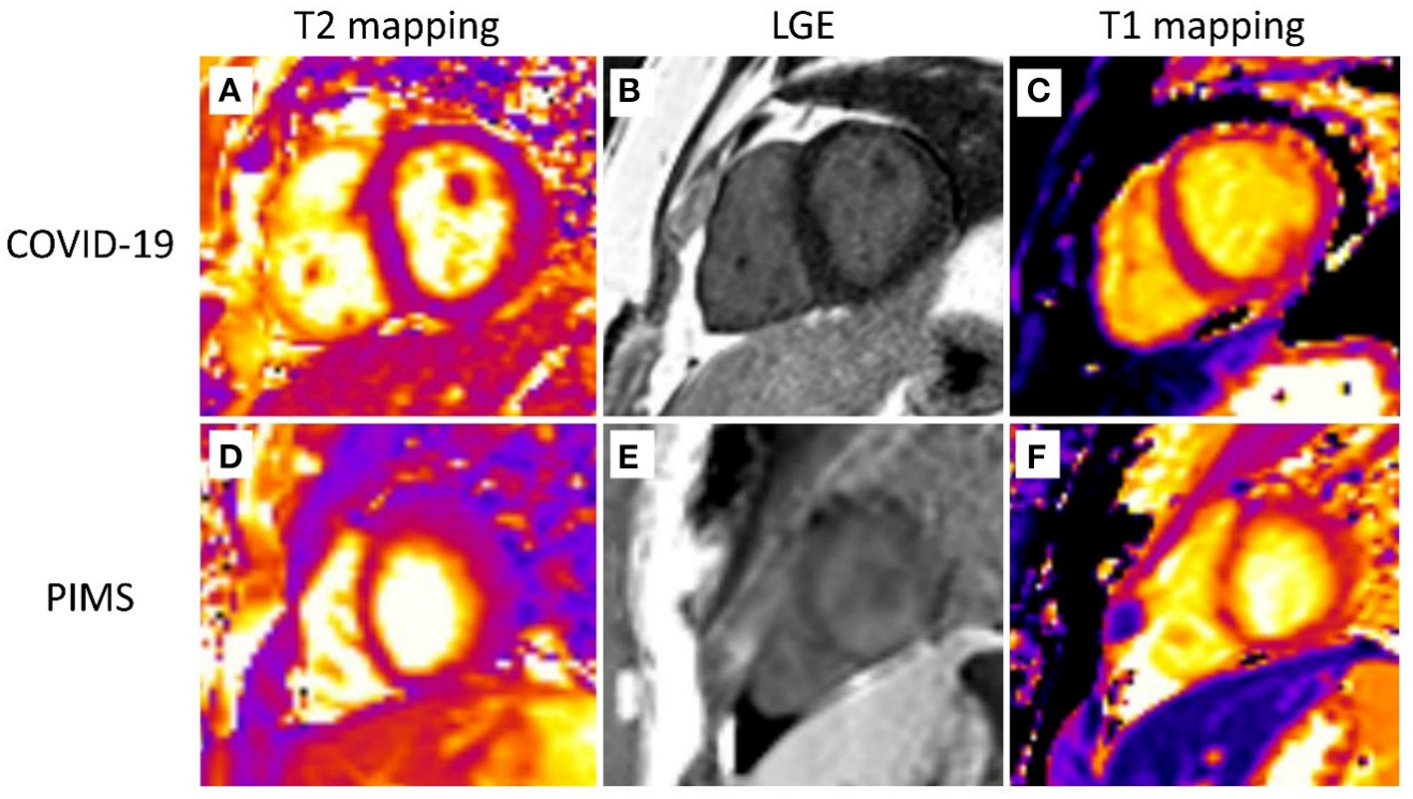
Cardiovascular magnetic resonance. Representative cardiovascular magnetic resonance images are shown. The first row depicts a 15-year-old patient after severe COVID-19 infection. T2 mapping **(A)**, LGE **(B)**, and T1 mapping **(C)** images display subepicardial and intramural LGE and prolonged T1 relaxation (up to 1,200 ms) along the anterior and anterolateral free wall of the LV with no signs of myocardial edema (normal T2 relaxation time-−45–47 ms). The second row shows images of an 11-year-old patient with pediatric multiorgan inflammatory syndrome (PIMS). T2 mapping **(D)**, LGE **(E)**, and T1 mapping **(F)** images display similar changes (subepicardial and intramural LGE and prolonged T1 relaxation up to 1,180 ms with normal T2 relaxation time) with a slightly different distribution—along the lateral and posterolateral free wall of the LV.

**Figure 2:**
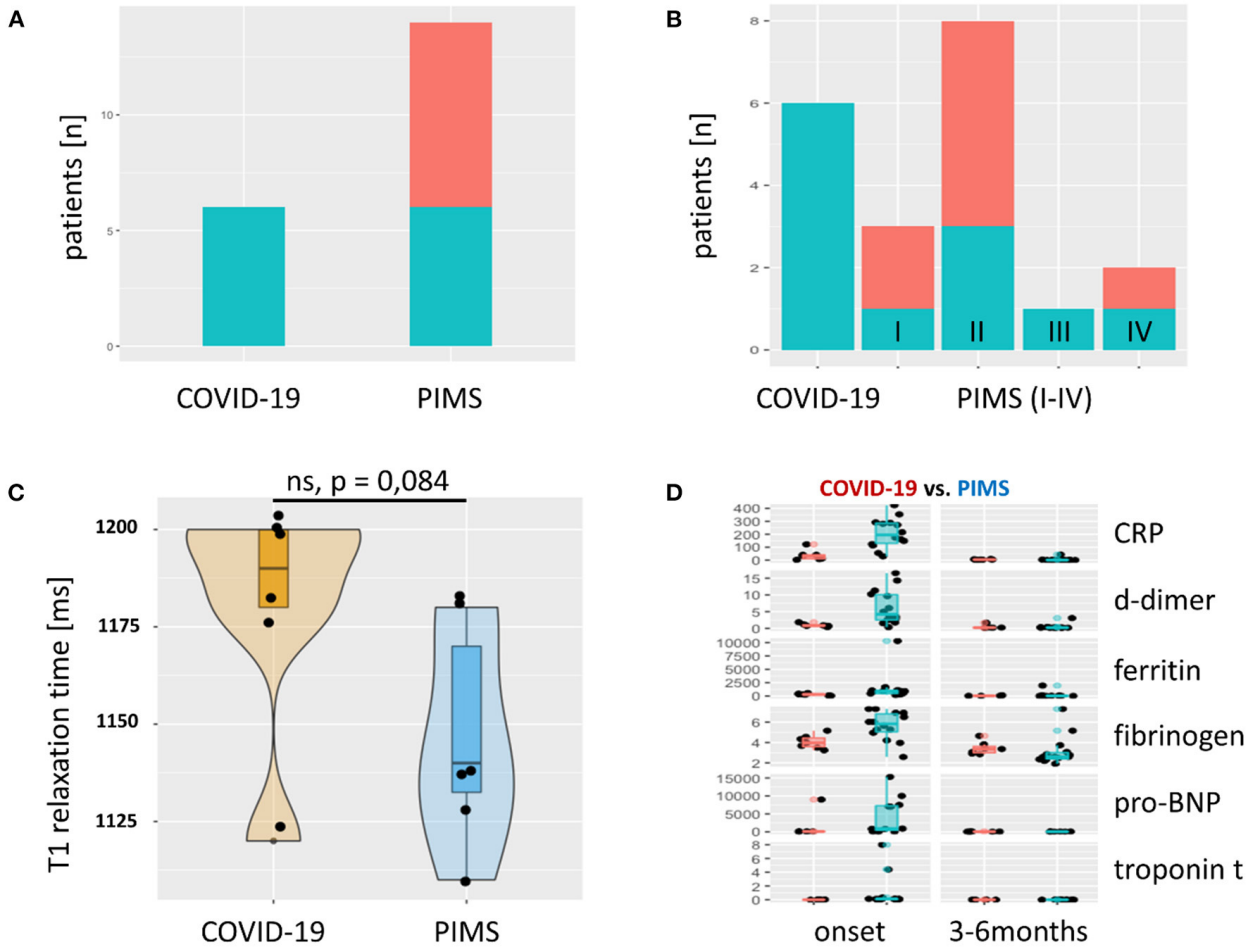
Late Gadolinium Enhancement (LGE) and T1 relaxation times and laboratory results. **(A)** The absolute frequency of LGE as bar graph. LGE (green) was found in every severe acute COVID-19 patient. In the group of patients with PIMS, we observed LGE in 6/14 (43%). Red = unaffected patients. In **(B)**, the PIMS group was divided into four subgroups: (I) Kawasaki-like without shock symptoms, (II) Kawasaki-like with shock, (III) PIMS without shock, and (IV) PIMS with shock. LGE could not be attributed to one of these four subgroups, although a trend toward group II Kawasaki with shock symptoms was seen. T1 relaxation time in areas of the myocardium with LGE is shown in **(C)** as violin plots with superimposed scatter plots. This shows a prolongation of relaxation time above the norm (< 1,100 ms, determined in 20 healthy subjects) in both groups. Patients with severe acute COVID-19 showed an even more pronounced prolongation of T1 relaxation time than patients with PIMS. There was no significant difference between the two groups (*p* = 0.084). Solid line = median. Markers of inflammation and myocardial damage of patients with COVID-19 (red) and PIMS (blue) at the time of disease onset and last visit are compared in **(D)**. All markers are more elevated in PIMS group but normalize completely until the last visit after 3–6 months. CRP, C-reactive protein [mg/L]; d-dimer [mg/L fibrinogen equivalent units], ferritin [ng/L], fibrinogen [g/L], pro-BNP, pro-brain natriuretic peptide [pg/mL], troponin t [ng/mL].

Cardiac MRI was obtained in all but one severe acute COVID-19 patient who aborted the examination due to a panic attack. A non-ischemic myocardial pattern of late gadolinium enhancement (LGE) was seen in 12 of 20 patients (Figure 1). All severe acute COVID-19 patients (6/6) were affected compared to only 43% (6/14) of PIMS patients (Figure 2A). There was no difference in the type of myocardial involvement between both groups (Figure 1). The left ventricle free wall was most involved with a distribution pattern starting at the epicardial surface and progressing to intramural zone. All areas (basal, mid, and apical) were affected with varying locations and grades.

**Figure 3:**
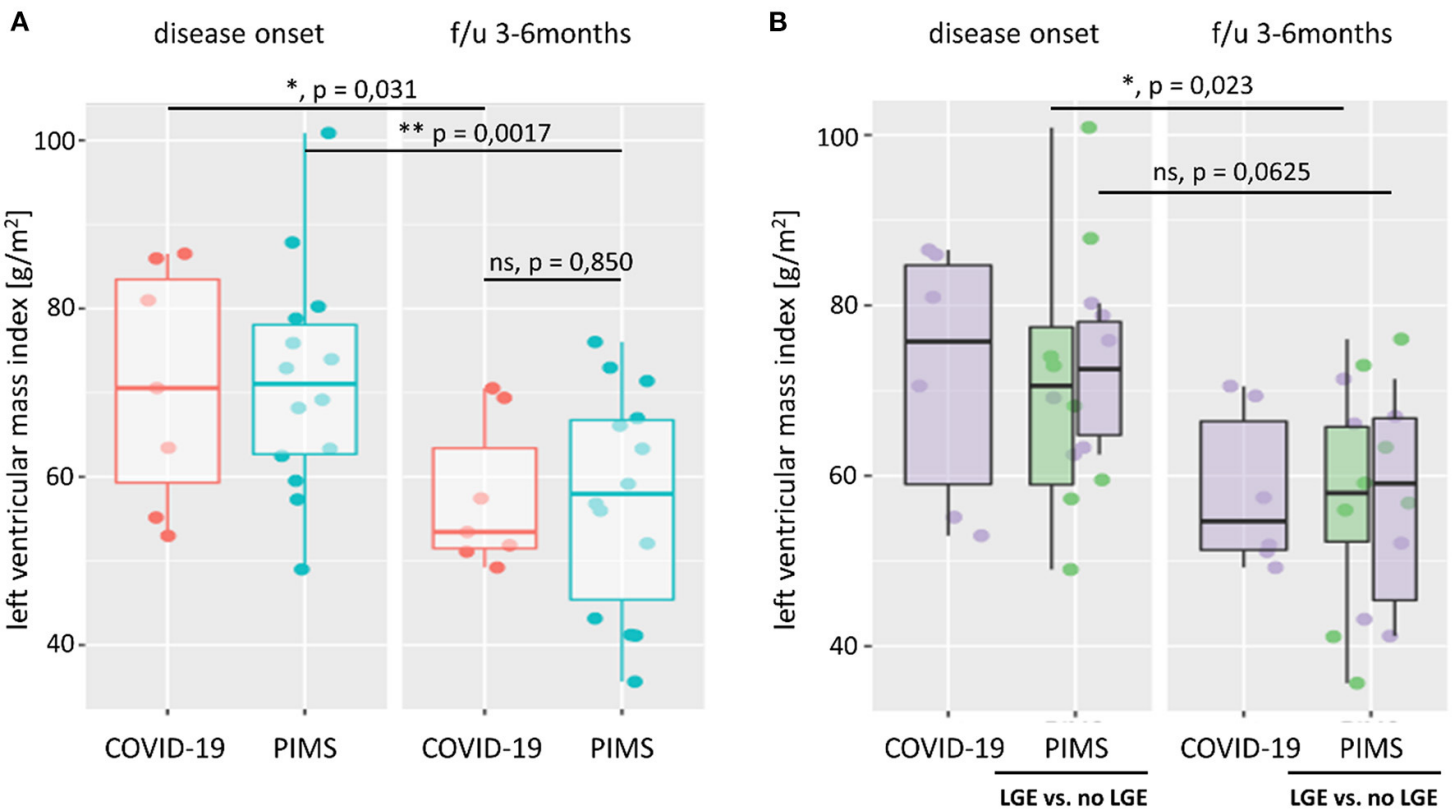
Left ventricular mass index. Left ventricular mass index (LVMI) was calculated from body surface area, and left ventricular mass was estimated using the Devereux equation. In **(A)**, we compare LVMI of patients with severe acute COVID-19 (red) or pediatric inflammatory multiorgan syndrome (PIMS, blue) at the time of disease onset and last follow-up (f/u) visit 3–6 months later. At both time points, there was no significant difference in LVMI between the two diagnoses (*p* = 0.971 at disease onset; *p* = 0.850 at f/u). However, both groups saw a significant reduction in LV mass (not shown) and LVMI at f/u. As shown in **(B)**, there was also no significant difference in LVMI between patients with PIMS with or without (green) late gadolinium enhancement (LGE) in cardiac MRI at the time of disease onset (*p* = 0.660) or f/u (*p* = 1). The difference n LVMI between non-LGE PIMS was more pronounced (*p* = 0.023) than in PIMS with LGE (*p* = 0.0625). See also Supplementary Figure 3 for LV *z* scores and connected dot plots.

Additionally, in 6 patients, limited LGE in the intraventricular septum was seen in basal and midseptum areas. This was observed in four children after severe COVID-19 and two with PIMS without significant differences between the two groups. The overall amount of affected areas in all patients was < 25% of the total myocardium (Figure 3). The distribution of LGE zones and, correspondingly, of prolonged T1 relaxation times revealed a non-ischemic inflammatory damage pattern. T1 relaxation times tended to be longer in patients with acute COVID-19 but did not differ significantly from patients with PIMS (Figure 2C). LGE could not be attributed to a certain subgroup of patients with PIMS as LGE was found in 2/3 patients with no KD-like PIMS and in 4/11 of KD-like PIMS (Figure 2B). The same was true for the 8 patients with coronary dilatation, where 4/8 showed LGE, data not shown.

The MRI data showed no differences in body weight-adjusted stroke volumes. End-diastolic volumes were all normal. Decreased cardiac contractility and EF were seen in only one severe acute COVID-19 patient. A decreased ejection fraction was found in one PIMS patient. Residual non-extensive edema was observed in one patient, accompanied by prolonged T2 relaxation time.

### 3.4. Therapy

All patients suffering from acute COVID-19 received corticosteroids [dexamethasone, mean dose 0.4 mg/kg/days (0.2–0.8 mg/kg/days)] or methylprednisolone [mean dose 1 mg/kg/days (0.9–2.0 mg/kg/days)], for a mean of 15 days, low molecular weight heparin (dose 1 mg/kg/days), and an antiplatelet agent. The patient who was put on mechanical ventilation also received treatment with remdesevir. All patients with PIMS were treated additionally with intravenous immunoglobulin 1–2 g/kg over 24 h after an average of 3 days after disease manifestation. In 2 patients, intravenous immunoglobulin was administered over 2–3 days due to severe circulatory shock.

## 4. Discussion

The evaluation of cardiac sequelae after severe acute COVID-19 in childhood and comparison with the better-characterized PIMS patients will help assess these patients' risk better and may improve recommendations for follow-up and overall outcome.

### 4.1. Late gadolinium enhancement persists in all patients after severe acute COVID-19 infection

In this study, we assessed the cardiac disease severity in patients with acute severe COVID-19 or PIMS to identify their risk profile and compare cardiac sequelae. Through a combination of cardiac MRI with readily available point-of-care examinations, such as ECG and echocardiogram, we found a significant but underreported risk for secondary cardiac damage in pediatric patients after acute severe SARS-CoV2 infection. This effect was mostly evident in MRI examination in the form of a non-ischemic pattern of LGE. This type of myocardial injury was reported in 30% of 47 adult patients with COVID-19 (19).

In most of our patients, a patchy mid-wall and sometimes subepicardial LGE was found. This distribution pattern is typically seen during or after myocarditis (20). In COVID-19 patients with MISC or isolated heart involvement, an identical MRI image with this myocarditis type is seen in all degrees of myocardial involvement (21).

Edema in the myocardium can be observed in myocarditis up to 6 months after the disease, depending on the severity of myocardial involvement (22).

Noteworthy, all patients (6/6) after severe acute COVID-19 were affected by LGE compared to only 35.7% (5/14) of patients with PIMS diagnosis. These data support previous studies showing that myocarditis may persist or even worsen despite normalized cardiac enzymes and inflammatory parameters (13, 23). Delayed LGE has been shown to be a good parameter for identifying this persistent disease activity despite normalizing biomarkers (23).

We also observed that at the time of pathological MRI findings, the previously elevated troponin T, pro-BNP and markers of inflammation had already normalized completely. Moreover, clinical laboratory results did not correlate with disease severity or occurrence of LGE. Two recent studies in younger adults with acute myocarditis but otherwise limited cardiovascular risk profile highlight the importance of this finding and its clinical implications (24, 25). Myocardial scarring detected by LGE persisted in 54% of the patients after 1 year (24). Moreover, it was shown that patients with mid-wall anteroseptal myocardial LGE were associated with a worse prognosis than other LGE patterns despite preserved LVEF (25). This matches our observations as all patients with acute COVID-19 showed normal LVEF but presented with LGE.

Our findings are in contrast with a recent report showing normal cardiac MRI in a cohort of 17 patients 2 months after severe acute COVID-19 or PIMS (26). In our view, these contradictory results can mainly be attributed to the use of LGE. The extent of cardiac involvement is primarily reflected through LGE changes in our data. In contrast, parameters such as ejection fraction and contractility are affected rarely and show no difference between PIMS and severe COVID-19. Our MRI study showed no difference in ventricular systolic stroke and diastolic volume. This observation exemplifies the superiority of cardiac MRI with LGE over native MRI or echocardiography for the follow-up for ongoing myocardial disease activity in COVID-19 patients (27).

### 4.2. Pathophysiology of myocardial disease

Most data on cardiac MRI currently available are from the subacute or convalescent phase and report high recovery rates of ventricular function (28, 38, 40) or mostly mild continuous cardiac dysfunction (29–32). Two recent studies report myocardial edema in 30–50% of patients (33); LGE was seen in 14% of patients (34). The frequently seen combination of myocardial edema and hyperemia without focal myocardial necrosis or fibrosis (36) indicates indirect myocardial injury due to severe acute inflammation rather than direct cardiomyocyte damage (37). Recent studies describe a myocarditis-type of PIMS (38, 39) that differs from the first described Kawasaki-type coronary artery disease PIMS (35, 41). Our results support these findings, as we saw a myocarditis-like distribution pattern in all patients with LGE. The decrease in LV mass in all patients further supports a long-lasting inflammatory component of cardiac involvement in SARS-CoV-2.

Interestingly, myocarditis after mRNA vaccines or PIMS demonstrates a similar pattern of myocardial injury (42, 43). This suggests that the general immune response to SARS-CoV2 may lead to a myocarditis-type inflammation, as seen in all our patients after severe acute COVID-19.

Although several studies using MRI for cardiac follow-up are currently conducted, many long-term recommendations for PIMS are being extrapolated from follow-up studies of children with Kawasaki disease and viral myocarditis. We recommend a close follow-up of all patients with PIMS or severe COVID-19. As cardiac involvement might occur despite normalized laboratory results and echocardiography, we recommend cardiac MRI after 3–6 months. In the case of abnormal findings, the examination should be repeated 9–12 months after disease onset.

### 4.3. Echocardiography, clinical findings, and implications for cardiac outcome

We attempted to find echocardiographic parameters that correlate with the MRI findings and thus be suitable and affordable in follow-up. In doing so, we hypothesized that myocardial edema is present at disease onset, increasing the LVMI. However, both groups showed an average left ventricular mass (score < 2). At follow-up, we observed a significant decrease in left ventricular mass and the resulting LVMI in both groups. We chose LVMI because both the pediatric *z*-scores for LV mass and the LV mass/height centiles are not validated for obese patients, as in our cohort. Normal *z*-scores for LV mass between +2 and −2 at disease onset indicate a normal LV mass without extensive edema. However, as MRI was not performed at disease onset, we cannot rule out the presence of subtle edema at this time.

According to the literature, a more pronounced cardiac involvement was seen in the patients diagnosed with PIMS (6, 41). Reduced cardiac function and mitral valve regurgitation both normalized in most patients within 6 months. We found more patients with relevant pathologies in the follow-up echocardiography than during the acute disease. This observation fits with the MRI data showing the persistence of myocardial involvement in all patients after acute COVID-19.

Echocardiographic findings after COVID or PIMS mostly consist of depressed LV function, coronary artery dilation or aneurysm, mitral regurgitation, and pericardial effusion (34, 35, 44, 45). Studies reporting a broad range of disease severity from mild infection to PIMS arrive at an overall rate of 30–40 percent for depressed LV function and 8–24 percent for CA abnormalities (6, 34, 41, 46, 47). Case series of only severely affected patients reported significantly higher rates of impaired LV function (~50–60%) and CA abnormalities (~20–50%) (4, 6, 47, 48). Our data show less acute functional involvement of the heart. We saw reduced LV function only in patients with PIMS. The same is true for transient MI, which also occurred in 7/8 cases of patients after PIMS.

A recent study that included 503 patients reports a favorable outcome for both conditions: LV function normalized within 30 days in 91% of patients, and nearly all patients with available data had normal LV EF at 90-day follow-up. In more than 75% of patients with CA aneurysms, they regressed to normal (*Z*-score < 2.5) within 30 days and in all patients up to 90 days (6).

Another study describing echocardiographic findings in 286 children with PIMS reported similar numbers: 34% of patients had depressed LV EF, 42% had mitral regurgitation, and 28% had pericardial effusions. Interestingly, of the 42 patients in whom cardiac MRI was performed in 42 patients, 34% of patients showed evidence of myocardial edema, but LGE was found in only 14% of patients (34). Several studies have reported abnormal strain patterns in patients with LV dysfunction (33, 35). Although strain analysis was not utilized in this study, we report a significant decrease in LV mass after both PIMS and COVID-19 despite normalized LV function (Supplementary material).

For the return to sports and physical activity, we follow the recommendations of the American Academy of Pediatrics of 2022, which recommend a sports break of 3–6 months after severe acute COVID-19 or PIMS. For patients who showed LGE on cardiac MRI after this period, we recommend resumption of light exercise in case of unremarkable physical examination, ECG, and no new onset of symptoms such as syncope, shortness of breath, chest pain, or palpitations.

### 4.4. Risk stratification of patients with severe acute COVID-19 or PIMS

The pattern of clinical presentation helps distinguish between acute COVID-19 and PIMS. Feldstein et al. (6) report that laboratory findings for cardiac and inflammatory markers correlate with the severity of cardiac involvement. However, identifying severe acute COVID-19 in pediatric patients is rather difficult due to the lack of a consensus definition for severe disease.

To minimize selection bias and identify possible subgroups at risk, we applied the already established PELOD2 score, which was developed to assess the severity of multiple organ dysfunction syndrome in the PICU on a continuous scale (15). However, we found the disease severity relatively underrepresented in the setting of severe acute COVID-19 and PIMS—partly by not considering ARDS in case of high flow oxygen therapy. A recent large multi-center suggests that early identification of children likely to progress to severe disease may be achieved close to admission using readily available parameters (49). It will be exciting to see if this method's predictive value might help identify children at high risk for cardiac involvement.

### 4.5. Limitations of this study and future directions

One of this study's limitations is the availability of MRI data only as a follow-up examination. Due to the recruitment of severe cases and the risk of anesthesia, cardiac imaging was not feasible in the acute phase of the respective disease. The comparison of only severely ill patients is another limitation of our study. To exclude the resulting selection bias, repeat studies should also include mild courses and compare patients of all severity levels. The comparatively small group size also hinders robust conclusions. More extensive studies are needed to validate our results and make well-founded recommendations. Another essential task for subsequent studies is to evaluate the clinical significance of ongoing myocardial disease activity and to report long-term follow-ups of these patients.

## 5. Conclusion

In summary, this study shows that children suffering from severe acute COVID-19 infection have a similar cardiac risk profile to patients with PIMS, despite differences in clinical presentation. Both groups showed persistent cardiac involvement and should therefore receive routine cardiological follow-up. Cardiac MRI should be performed during the follow-up of severe acute COVID-19 or PIMS patients, as most of them had delayed late enhancement, even after laboratory and echocardiographic findings had normalized.

## Data availability statement

The original contributions presented in the study are included in the article/Supplementary material, further inquiries can be directed to the corresponding author.

## Ethics statement

The studies involving human participants were reviewed and approved by Plovdiv Medical University Scientific Ethics Committee. Written informed consent to participate in this study was provided by the participants' legal guardian/next of kin.

## Author contributions

LC-B, MB, and KG conceived and designed the analysis, collected the data, contributed data or analysis tools, performed the analysis, and wrote the paper. DF conceived and designed the analysis, performed the analysis, and wrote the paper. SA, SS, IP, ZH, IN, AS, and KK collected the data and wrote the paper. II conceived and designed the analysis, contributed data or analysis tools, performed the analysis, and wrote the paper. All authors contributed to the article and approved the submitted version.
