# Supplementary material for: Cardiac MRI with late gadolinium enhancement shows cardiac involvement 3–6 months after severe acute COVID-19 similar to or worse than PIMS
Source: Front Cardiovasc Med. 2023 Jan 25;10:1115389. doi: 10.3389/fcvm.2023.1115389 (PMC9905637; doi:10.3389/fcvm.2023.1115389)
Supplement: Supplementary file 1 [file Data_Sheet_1.docx]

Supplementary Material

Cardiac MRI with late gadolinium enhancement shows cardiac involvement 3-6 months after severe acute COVID-19 similar to or worse than PIMS

Lyubov A. Chochkova-Bukova^1, #, *^, Dominik Funken^2, #^, Mila Bukova^3, #^, Kamelia Z. Genova^4^, Sadika Ali^1^, Snezhana Stoencheva^1^, Ivanka N. Paskaleva^1^, Zeira Halil^1^, Ivelina Neicheva^1^, Anastasia Shishmanova^1^, Kristina Stefanova Kelly^1^, Ivan S. Ivanov^1^

*** Correspondence:** Lyubov A. Chochkova-Bukova: lyubov.chochkova@mu-plovdiv.bg

#

# Supplementary Figures

**1.1 Supplementary Figure 1 Independence of laboratory results and late gadolinium enhancement (LGE) in cardiac MRI**

**
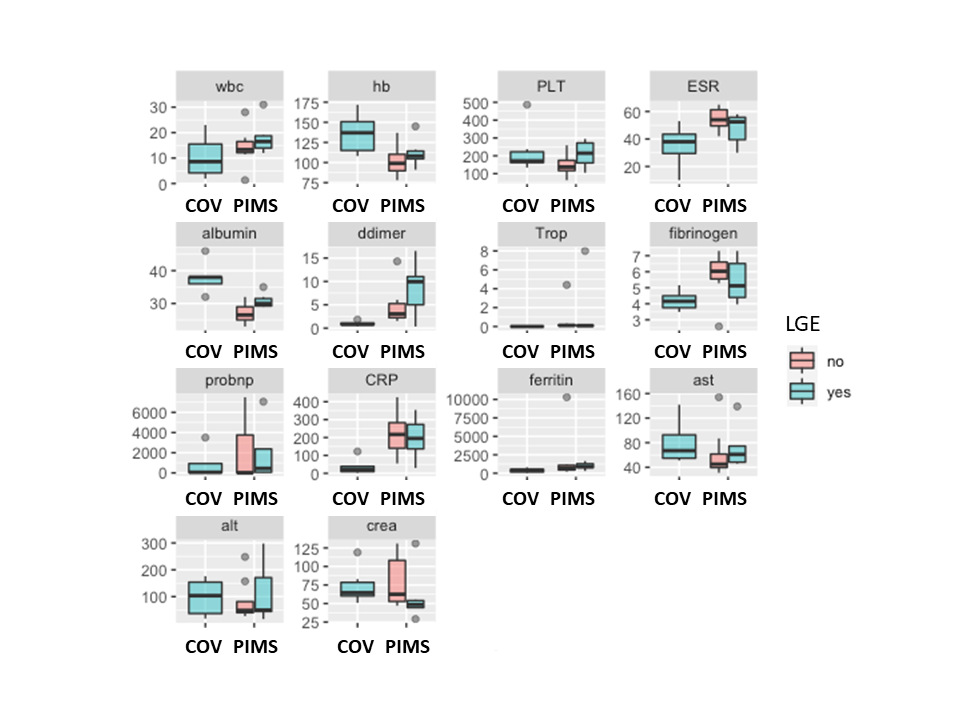
**

**Legend Suppl. Fig. 1**

The laboratory results of patients with severe acute COVID-19 (“COV” = red) or PIMS (green) are shown. We found no correlating biomarker profile for the occurrence of LGE in cardiac MRI. Patients with severe acute COVID-19 are compared to patients with PIMS (with (green) or without LGE (red). All differences between groups were non-significant. Wbc = white blood cell count [tsd/µL], Hb = hemoglobin [g/L], PLT = platelets [tsd/µL], ESR = erythrocyte sedimentation rate [mm/hr], albumin [mg/L], d dimer [mg/L fibrinogen equivalent units], Trop = troponin t [ng/mL], fibrinogen [g/L], probnp = pro-BNP [pg/mL], CRP = C-reactive protein [mg/L], ferritin [ng/L], AST = aspartate aminotransferase [IU/L], ALT = alanine aminotransferase (GPT) [IU/L], crea = creatinine [µmol/mL].

**Supplementary Figure 2 Late gadolinium enhancement, cardiovascular damage, and disease severity**


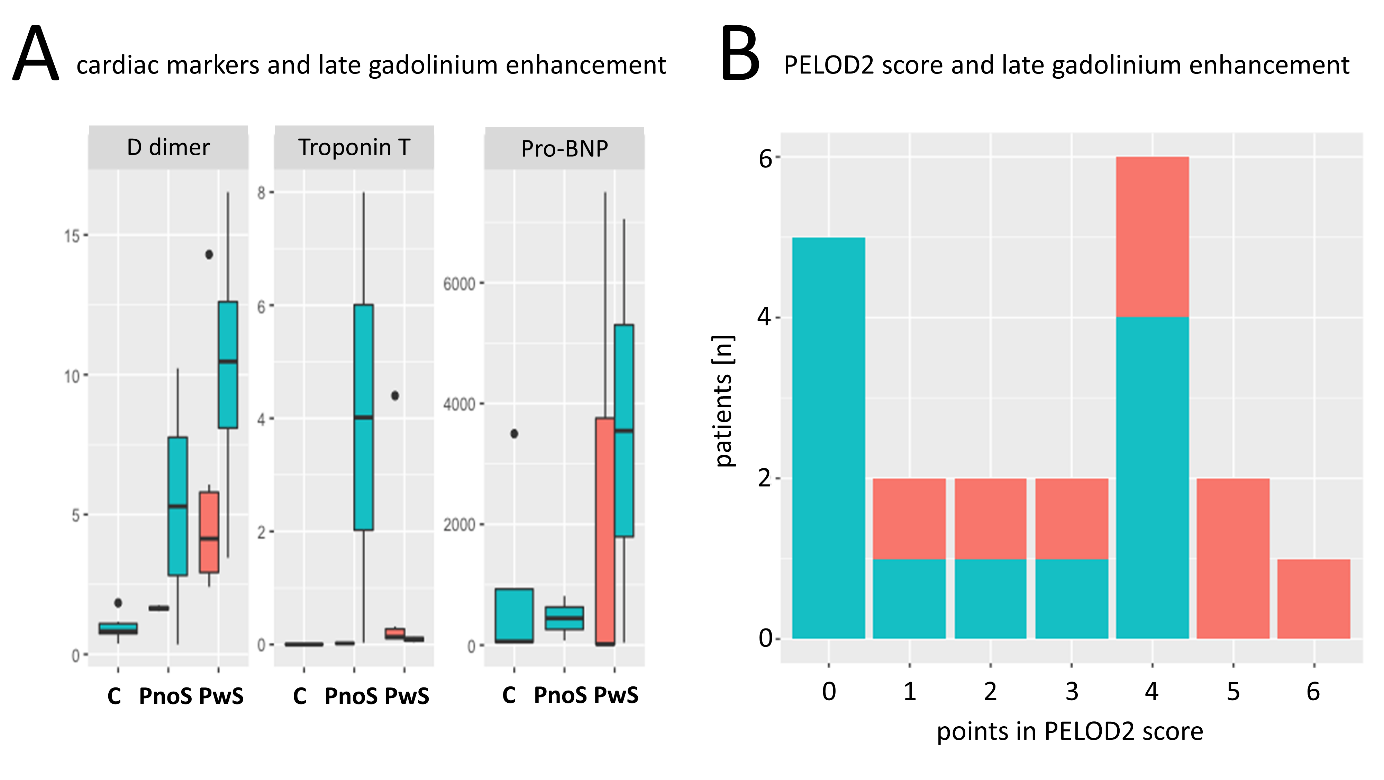


**Legend Suppl. Fig. 2**

The occurrence of LGE on cardiac MRI in our cohort did not correlate with markers of myocardial damage, cardiovascular shock symptoms (A), or disease severity on the PELOD2 score (B). In A, we divided patients into three groups: severe acute COVID-19 (C) and PIMS without (PnoS) or with cardiovascular shock symptoms (PwS). The serum levels of d-dimers, troponin t, and pro-BNP at disease onset are shown in blue for patients with LGE or red for patients without LGE. No specific marker pattern was found. In B, we plotted the occurrence of LGE in correlation with the disease severity according to the PELOD2 score. Patients with LGE are shown in blue, and those without LGE in red. There was no correlation between PELOD2 score result or LGE.

**Supplementary Figure 3 Decrease of left ventricular mass after acute COVID-19 and PIMS.**


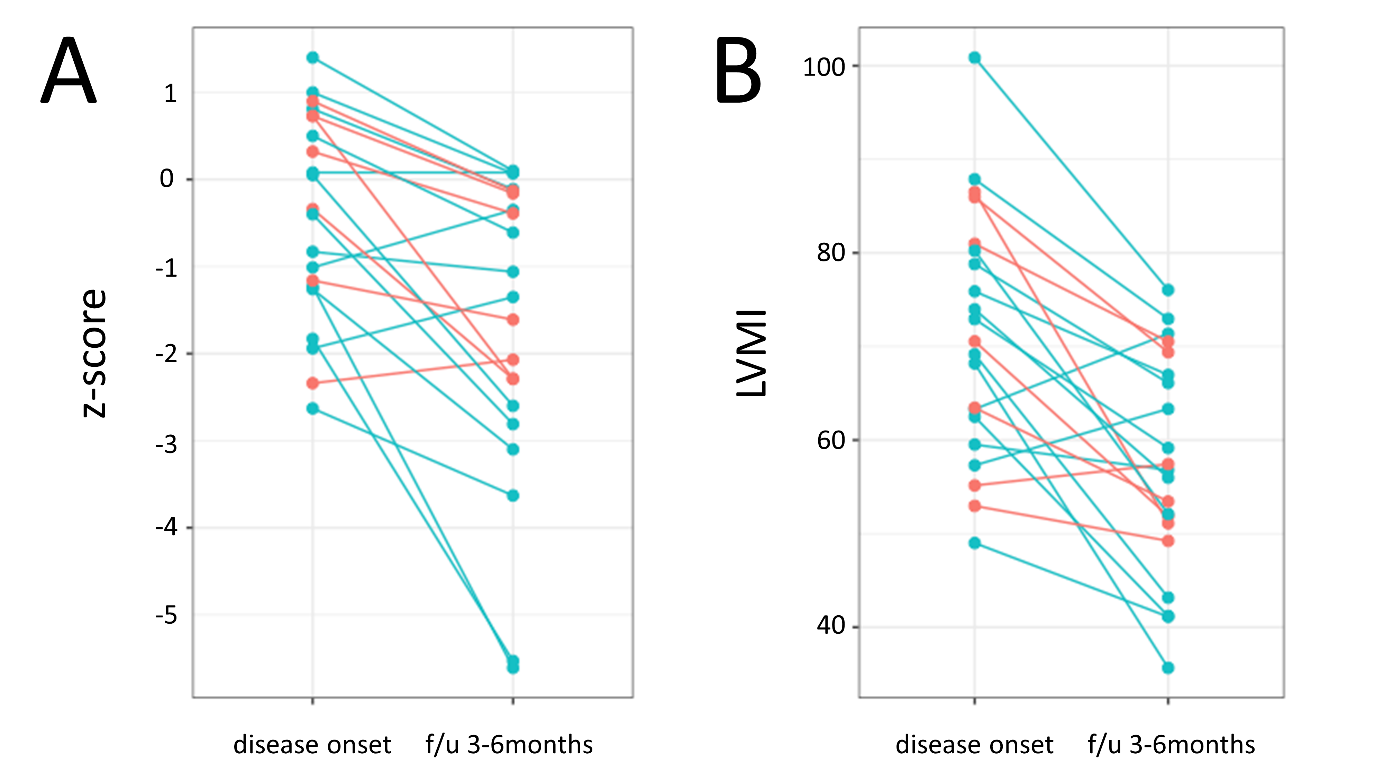


**Legend Suppl. Fig. 3**

Patients with severe acute COVID-19 are shown in red, PIMS in blue. A shows echocardiographically measured left ventricular (LV) mass development as z-scores. At disease onset, LV mass is within the normal range in almost all patients (z score (-2 to 2). The same course is shown in B as left ventricular mass index (LVMI). Direct comparison shows a general reduction in LV mass for both groups for both z-scores and LVMI, see also Figure 4.
